# Nanoparticle-Mediated Combination Therapy: Two-in-One Approach for Cancer

**DOI:** 10.3390/ijms19103264

**Published:** 2018-10-20

**Authors:** Sangiliyandi Gurunathan, Min-Hee Kang, Muhammad Qasim, Jin-Hoi Kim

**Affiliations:** Department of Stem Cell and Regenerative Biotechnology, Konkuk University, Seoul 05029, Korea; pocachippo@gmail.com (M.-H.K.); qasimattock@gmail.com (M.Q.)

**Keywords:** liposomes, polymeric nanoparticles, dendrimers, carbon nanoparticles, graphene oxide nanocomposites, metallic nanoparticles, anticancer drug, combination therapy

## Abstract

Cancer represents a group of heterogeneous diseases characterized by uncontrolled growth and spread of abnormal cells, ultimately leading to death. Nanomedicine plays a significant role in the development of nanodrugs, nanodevices, drug delivery systems and nanocarriers. Some of the major issues in the treatment of cancer are multidrug resistance (MDR), narrow therapeutic window and undesired side effects of available anticancer drugs and the limitations of anticancer drugs. Several nanosystems being utilized for detection, diagnosis and treatment such as theranostic carriers, liposomes, carbon nanotubes, quantum dots, polymeric micelles, dendrimers and metallic nanoparticles. However, nonbiodegradable nanoparticles causes high tissue accumulation and leads to toxicity. MDR is considered a major impediment to cancer treatment due to metastatic tumors that develop resistance to chemotherapy. MDR contributes to the failure of chemotherapies in various cancers, including breast, ovarian, lung, gastrointestinal and hematological malignancies. Moreover, the therapeutic efficiency of anticancer drugs or nanoparticles (NPs) used alone is less than that of the combination of NPs and anticancer drugs. Combination therapy has long been adopted as the standard first-line treatment of several malignancies to improve the clinical outcome. Combination therapy with anticancer drugs has been shown to generally induce synergistic drug actions and deter the onset of drug resistance. Therefore, this review is designed to report and analyze the recent progress made to address combination therapy using NPs and anticancer drugs. We first provide a comprehensive overview of the angiogenesis and of the different types of NPs currently used in treatments of cancer; those emphasized in this review are liposomes, polymeric NPs, polymeric micelles (PMs), dendrimers, carbon NPs, nanodiamond (ND), fullerenes, carbon nanotubes (CNTs), graphene oxide (GO), GO nanocomposites and metallic NPs used for combination therapy with various anticancer agents. Nanotechnology has provided the convenient tools for combination therapy. However, for clinical translation, we need continued improvements in the field of nanotechnology.

## 1. Introduction

**Figure 1:**
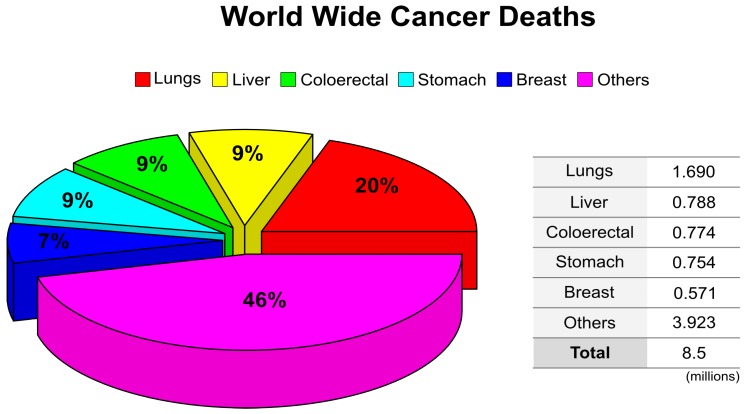
The worldwide percentage distribution of cancer types (https://www.cancer.org/research/cancer-facts-statistics/all-cancer-facts-figures/cancer-facts-figures-2018.html, Accessed on 11 July 2018).

Cancer is a public health problem and one of the leading causes of death worldwide, claiming annually more than 8 million lives [1,2]. The International Agency for Research on Cancer (IARC) reported there were 8.2 million cancer deaths worldwide in 2012 and predicted it will increase up to 13 million by 2030 (Figure 1).

Cancer deaths have increased at an alarming rate in both developed as well as developing countries (https://www.cancer.org/research/cancer-facts-statistics/all-cancer-facts-figures/cancer-facts-figures-2018.html, Accessed on 11 July 2018). Currently, cancers are being treated with a variety of treatments including chemotherapy, hormone therapy, radiation, surgery, immune therapy and targeted therapy [3,4]. Therefore, further interventions are necessary to lower the rate and manage the disease by implementing various strategies, including new treatment methods. Although various therapeutic modalities are available, it is essential to identify the most effective treatment. Although the conventional therapies have improved patients’ survival, they also have several limitations [5], such as non-specific targeting, drug resistance, excessive toxic effects and undesired side effects. Among several new therapeutic approaches, nanotechnology in cancer plays several roles including the detection and treatment of cancer, identification of biomarkers, understanding of cancer progress and guiding the development of new diagnostics and imaging agents [6].

**Figure 2:**
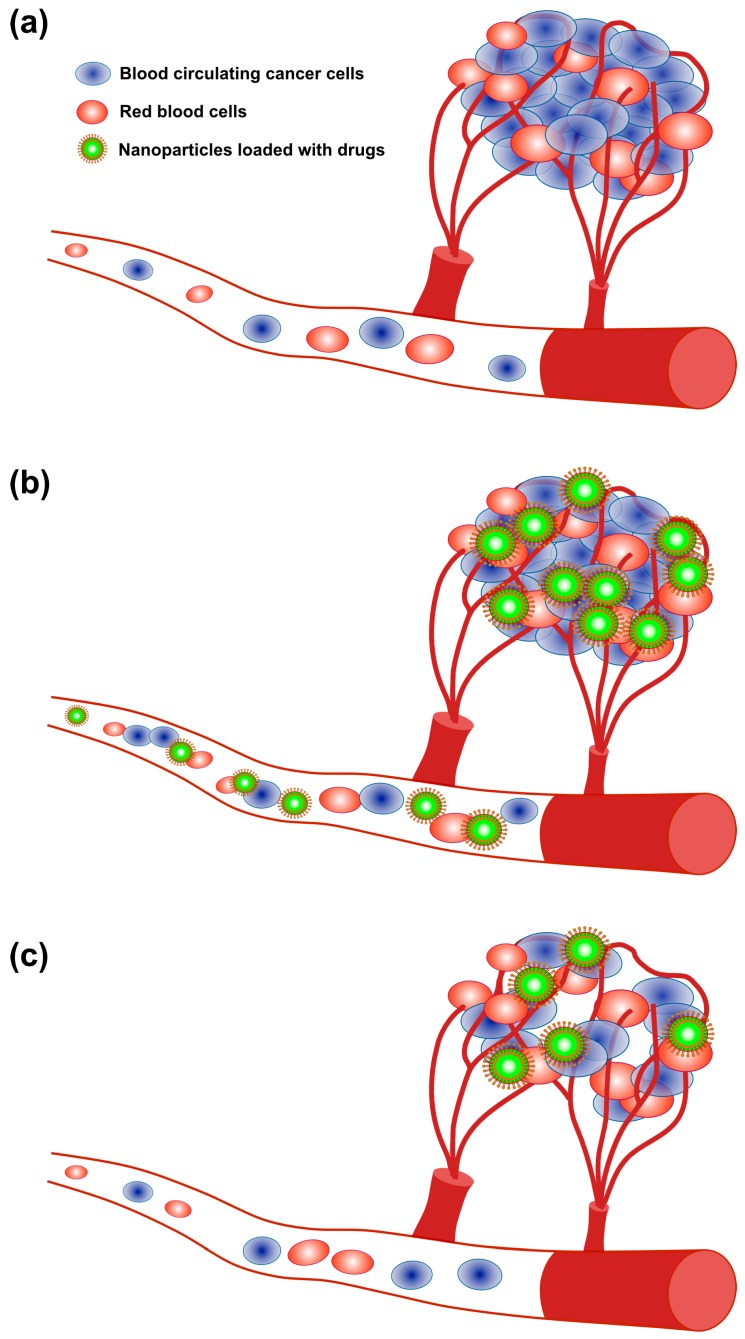
The angiogenesis process and treatment of tumors via nanoparticle delivery. (**a**) Angiogenesis; (**b**) Drug delivery through NPs; (**c**) Tumor reduction.

Angiogenesis is a vital, complex, regulated and integrated sequence of events involving formation of new blood vessels from the pre-existing ones, which is a hallmark of tumor development and metastasis [7,8]. Angiogenesis is an inevitable process for tumor growth, invasion and metastatic dissemination [7]. Physiological angiogenesis of proliferating endothelial cells in the ovaries, uterus and placenta is limited, whereas pathological angiogenesis is vigorous in cancer and in the wound-healing process [9], where it is critically controlled by the level of pro-angiogenic and anti-angiogenic factors and coordination among different types of cells including macrophages, endothelial cells and pericytes [7,8,10]. Angiogenesis is an essential process in cancer and in diabetic retinopathy, in which the cells are characterized by abnormal vasculature structure and functions such as tortuous, dilated, saccular vessels and high permeability [7,8,10,11]. The abnormal vascular structure leads to increased levels of vascular endothelial growth factor (VEGF) and decreases the effectiveness of cytotoxic chemotherapy [8,12]. Among several growth factors, VEGF plays a key role in regulation, both in normal and cancer cells, promoting endothelial cell migration, proliferation and capillary tube formation. The elevated level of VEGF is considered a major factor for tumor formation and abnormal vessel formation [12]. Controlling tumor angiogenesis is an integral part of the host defense system and is regulated by angiogenesis inhibitors such as endostatin and thrombospondin-1; the imbalance between inhibitors and growth factors leads to angiogenesis [13,14,15]. Numerous modalities have been developed for angiogenesis-related diseases to prevent new vessel formation in tumors and diabetic retinopathy, including: preclinical and clinical trials have tested VEGF antibodies as a therapy [16]; an angiogenesis inhibitor is able to kill highly proliferative tumor cells by depriving the tumors of nutrients and oxygen [17]; bevacizumab, an anti-VEGF antibody, significantly increases overall survival of patients with conventional chemotherapeutic agents for different type of cancers [18]; and Sunitinib, an anti-angiogenic drug that inhibits the VEGF receptor (VEGFR) tyrosine kinase and Sorafenib, an anti-angiogenic RTKI, show superior activity in patients with renal cell carcinoma when compared to the standard of care interferon-α treatment [19,20] (Figure 2).

Although various technologies have been developed to aid in understanding cancer biology and anticancer therapeutics, the mortality rates for many common cancers have improved only slightly in the past two decades. Therefore, innovative approaches are needed to improve the efficacy and the therapeutic index of anticancer therapy [21]. Targeting treatments against solid tumors by a variety of modalities leads to dramatic regressions; however, the effect is only short-term due to development of resistance to the therapy. Therefore, the use of multiple therapeutic anticancer agents in combination with NPs seems to be a promising strategy to treat drug-resistant cancer.

Nanomaterials-based cancer therapy plays an important role in increasing the therapeutic efficiency against cancer by utilizing a combination of nanomaterials and chemotherapeutic agents; combination therapy has been shown to increase tumor control and reduce undesired side effects by improving the pharmacokinetics and targeted delivery of the drug payloads [21,22]. Combination therapy seems to be a standard practice in conventional chemotherapy to overcome cross-resistance and achieve synergistically enhanced therapeutic outcome without significantly increasing toxicities. Generally, monotherapy is based on usage of a single drug, which is not sufficient to bring about tumor regression. With combination chemotherapy, the synergistic effect of two (or more) agents targeting different disease pathways, genes, or cell-cycle checkpoints in the cancer process are leveraged to raise the chances of eliminating cancer [23]. Therefore, combination therapy is a suitable and alternative mode of therapy and a promising approach to enhance the efficacy of cancer treatment. NP-mediated combination therapy would deliver multiple therapeutic agents with different physico-chemical and pharmacological properties in single NPs. NPs are able to maintain the optimized synergistic drug ratio in a single carrier all the way to the point of intracellular uptake by the target cancer cell. In practice, combination chemotherapy (“two-in-one” approach) results in a better response and an improved survival compared with single-agent therapy for clinical and preclinical investigations [23]. During the last two decades, nanotechnology has received significant attention and has contributed to clinical therapeutics. Particularly, the advances in biocompatible nanoscale drug carriers, such as liposomes and polymeric NPs, have enabled safer and more efficient delivery of a myriad of drugs.

**Figure 3:**
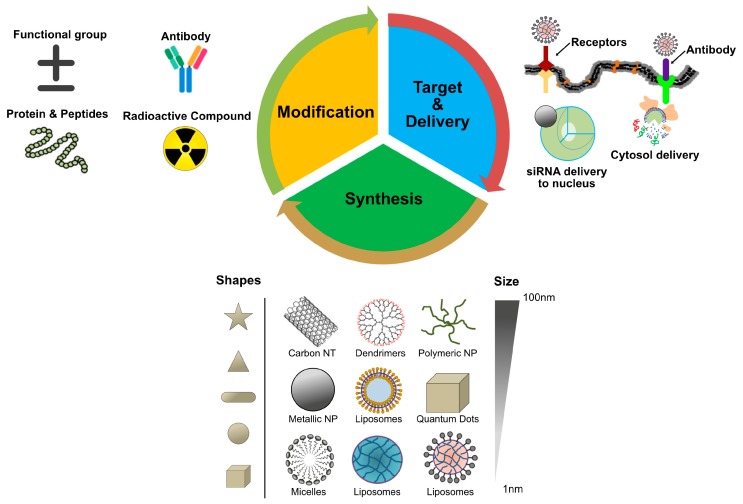
Central dogma of nanoparticles targeting in cancer cells.

NPs are promising drug carriers because of their ability to deliver hydrophobic and/or hydrophilic drug molecules, peptides, small molecule drugs, or antibodies to the tumor site with minimum toxicity to surrounding tissues; this safe delivery is based on their penetration capacity [24]. The potential anticancer properties of chemotherapeutic agents’ ability and delivery have been explored using various kinds of NPs, including particularly liposomes, polymeric NPs, PMs, dendrimers, carbon NPs, polymer-drug conjugates, nanoemulsions, CNTs, quantum dots and inorganic NPs (Figure 3). Recently, theranostic nanocarriers have shown much interest for various treatment due to their ability to execute simultaneous functions at targeted diseased sites. Nanotheranostic carriers composed of metallic or magnetic nanoparticles. In addition, we explained not only combinatorial aspects of nanoparticles but also the mode of action individual cytotoxic agent. Herein, we explained not only combinatorial aspects of nanoparticles but also the mode of action individual cytotoxic agent.

The use of NPs provides several advantages, such as decreasing drug-related toxicity and MDR by targeting various metabolic and physiological characteristics, improving plasma half-life, bio-availability and bio-distribution of drugs, enhanced permeability and retention (EPR) effect and sustained controlled drug release [25,26,27]. Recently the efficacy of chemotherapeutic agents was enhanced by RNA interference (RNAi)-mediated technology such as siRNA and miRNA, which can selectively knock down the expression from genes of interest or the effectiveness of drugs. Therefore, combining this technology with that of NPs could be a promising strategy for cancer therapy [28].

The merits of combination therapy include the use of chemotherapeutic agents and NPs at lower doses, elimination of undesired cytotoxic effects and increased efficacy, which presents a promising approach for cancer research [29]. In practice, combination chemotherapy results in a better response and improved survival compared with single-agent therapy. To overcome the complications of multiple drugs with dissimilar pharmacokinetics and bio-distribution due to different rates of metabolism within the body, nanoformulations can avoid such limitations by carrying multiple therapeutic agents, each with different physico-chemical properties and pharmacological behaviors; NPs are able to maintain the optimized synergistic drug ratio in a single carrier to the point of intra-cellular uptake into the target cancer cell. Therefore, combination therapy is a suitable and alternative approach for cancer therapy [30,31]. This review has been focused on various criteria such as: (a) worldwide cancer statistics; (b) process of angiogenesis in cancer; (c) targeting of cancer cells by nanoparticles; (d) type of various nanoparticles used for cancer therapy; (e) effect of nanoparticles on cancer; (f) effect of single anticancer drug on cancer; (g) effect of combination of anticancer drug and nanoparticles; (h) state of the art of combination therapy and limitations; (i) finally, conclusions and future perspectives on combination therapy.

## 2. Types of NPs Used for Combination Therapy

### 2.1. Liposomes

The emergence of nanotechnology has made a significant impact on clinical therapeutics in the last two decades. Liposomes and polymeric nanoparticles have been used as nanoscale drug carriers for more efficient and safer delivery of innumerable drugs. Among different varieties of NPs, the liposomes are the most established drug-delivery vehicle, with many clinical products available to date. Liposomes consist of amphiphilic lipid molecules that assemble into bi-layered spherical vesicles [32,33]. Liposome-mediated drug delivery is an improvement due to their efficiency, bio-compatibility, non-immunogenicity, enhanced solubility of chemotherapeutic agents and their ability to encapsulate a wide array of drugs [34]. Furthermore, liposomes have shown tremendous therapeutic potential as carriers for payloads and for delivery to targeted sites [35]; liposome-mediated drug delivery systems improve the pharmacokinetic and pharmacodynamic profiles of the therapeutic payload, promote controlled and sustained release of drugs and exhibit lower systemic toxicity compared with the free drug [35]. For instance, polyethylene glycol (PEG)-coated liposome-mediated drug delivery increased the in vivo circulation half-life of liposomes from a few hours (h) to approximately 45 h [36]. At present, different liposomal products being used for cancer treatment include Doxil, DaunoXome^®^, DepoCyt^®^ and ONCO-TCS, which are liposomal formulations of doxorubicin (DOX), daunorubicin, cytarabine and vincristine, respectively [37,38,39,40,41,42]. Lammers et al. (2009) demonstrated that simultaneously delivering multiple chemotherapeutic agents to tumors in vivo using HPMA-based polymer-drug conjugates carrying 6.4 weight % of gemcitabine, 5.7 weight % of DOX and 1.0 molar % of tyrosinamide. The resulting construct, poly(HPMA-co-MA-GFLG-gemcitabineco-MA-GFLG-DOX-co-MA-TyrNH2), was shown to be effective at killing cancer cells under in vitro conditions; additionally, circulating time was prolonged and localization efficiency to inhibit tumor growth was very selective [43]. Regarding liposome-mediated therapy, various strategies have been adopted for passive targeting of liposomes to the tumor sites by using PEG and active targeting of cancer cell surface receptors, FRs, TfR and EGFRs and the tumor microenvironment including VEGF, VCAM, matrix metalloproteases, αβ-integrins and surface grafting of liposomes with aptamers [34]. Use of nanotechnology could improve the pharmacokinetics and reduce side effects associated with drugs.

**Figure 4:**
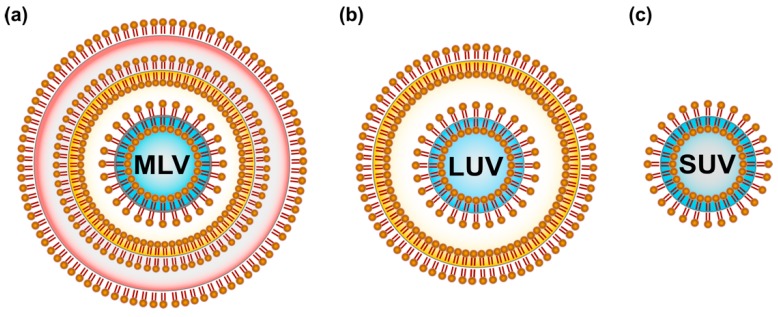
Classification of liposomes based on the lamellarity: (**a**) Multilamellar vesicles (MLV) are composed of many lipid bilayers and range from 1–5 µm in size; (**b**) Large unilamellar vesicles (LUV) are in the size range of 100–250 nm with a single lipid bilayer; (**c**) Small unilamellar vesicles (SUV) consist of a single phospholipid bilayer surrounding an aqueous phase with a size range of 20–100 nm.

Lipids were the first self-assembled materials used for drug delivery, mainly in the form of micelles and liposomes [44]. Liposomes are classified on the basis of size and the number of phospholipid membrane layers [45,46], such as multilamellar vesicles (MLV) with an average size between 1 and 5 µm, large unilamellar vesicles (LUV) in the size range of 100–250 nm with a single lipid bilayer and small unilamellar vesicles (SUV) consisting of a single phospholipid bilayer surrounded by an aqueous phase with a size range of 20–100 nm [47] (Figure 4).

Liposomes are the most established drug-delivery vehicle, with many clinical products to date. Liposomes consist of amphiphilic lipid molecules that assemble into bi-layered spherical vesicles [48]. The most frequently used building materials for liposomes are phosphatidylethanolamine (PE), phosphatidylcholine and cholesterol, which are able to incorporate into liposomal membranes easily; these materials enhance liposomal stability and rigidity, delivery pharmacokinetics and the efficacy of bioactive agents such as drugs, proteins, or oligonucleotides. Other favorable characteristics include that liposomes are non-toxic, non-invasive and can deliver hydrophilic and/or lipophilic substances. Liposomes play an important role in drug delivery for cancer treatment.

**Figure 5:**
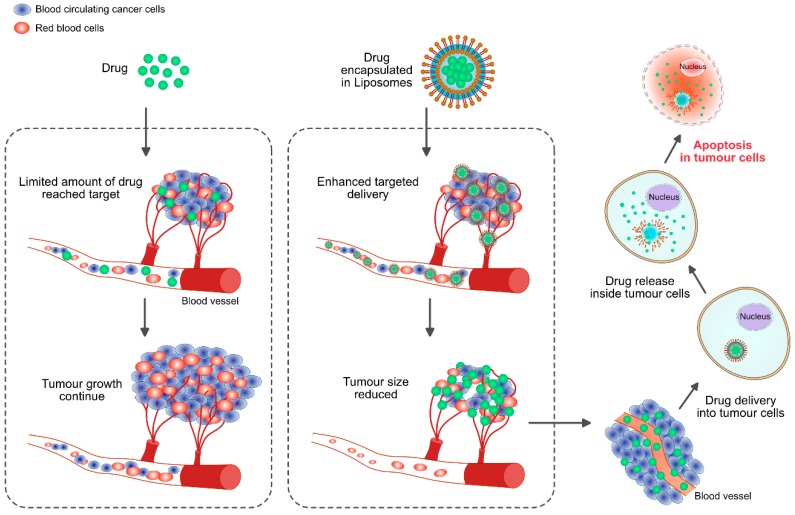
Drug delivery with and without Nanoparticles. Nanoparticle based targeted drug delivery enhanced the antitumor activity due to enhanced and sustained release of drug.

The size of NPs is crucial for successful delivery into melanoma tumors. Vesicles of <100 nm have reduced uptake into liver tissue, while vesicles >100 nm are prone to rapid clearance rates by the mononuclear phagocytic system [49] (Figure 3). Therefore, surface modification is a crucial factor for drug delivery. It was to address this issue that Gabizon et al. (1994) designed surface-modified liposomes with PEG [50]. In formulating liposomes to enhance nanovesicle content unloading, not only is the availability of a variety of lipids a critical factor but also heating and light are important. For example, thermosensitive NPs have been developed for DOX that enhance cytotoxicity about 20 times more in vitro in Lewis lung carcinoma (LLC) cells at 42 °C compared to 37 °C [51]. Liposomal-mediated benzoporphyrin derivative monoacid (available as verteporfin) increases the efficacy of photodynamic therapy of pigmented choroidal melanoma in tumor-bearing rabbits [52]. The combination of radiotherapy (XRT) with stealth liposomal DOX is feasible for the reduction of tumors in non-small-cell lung cancer and head and neck cancer [53]. Bcl-2 antisense oligodeoxynucleotide (AS ODN) G3139 combined with free DOX or sterically stabilized liposomal DOX was able to delay the growth of MDA435/LCC6 cells compared with control ODN-treated animals by reducing expression of Bcl-2, which increases the degree of antitumor activity. These results suggest that the combination of Bcl-2 AS ODN with liposomal formulations of anticancer drugs such as DOX can improve treatment [54]. Safra et al. (2001) demonstrated that pegylated liposomal DOX seems to be an effective drug when it is given as a secondary therapy to patients with epithelial ovarian carcinoma [55]. Similarly, liposomal encapsulation of daunorubicin specifically targeted to the tumor site with decreased toxicity, whereas the combination of daunorubicin with ara-C has significant anti-leukemia activity with acceptable toxicity in patients with refractory or recurring acute myelogenous leukemia [56]. DOX has played an important role in the treatment of patients with breast cancer for many decades and it is known that DOX has excellent antitumor activity in the metastatic, neoadjuvant and adjuvant settings. When used as liposomal DOX or pegylated liposomal DOX, it shows high efficacy and tolerability in patients with locally advanced breast cancer [57]. Combination of LErafAON with DOX or paclitaxel (PTX) led to significantly enhanced antitumor activity in all the tumor types, compared with LErafAON-alone or chemotherapeutic agent-alone treated groups, through inhibition of Raf-1 expression in tumor tissue [58] (Figure 5).

**Figure 6:**
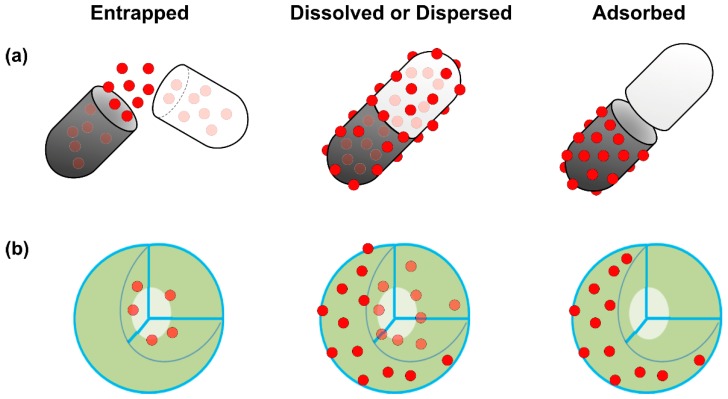
Encapsulation mechanism models: drug entrapped in, dissolved in, or dispersed within and adsorbed on: (**a**) nanocapsules and (**b**) nanospheres [67].

Liposomes (PC/CH/DMPE) sterically stabilized by PEG (PEG-DSPE) or equipped with the carbohydrate ligand sialyl Lewis(X) (conjugated to PEG-DMPE or to DMPE as an anchor) enhanced aggregation. In addition, the surface-modification increased uptake from 8.3% (only tumor cells) to 30.2% in HT29 cells [59]. When ovarian and endometrial cancer cells were transfected with the herpes simplex virus thymidine kinase (*HSV-tk*) gene and treated with ganciclovir (GCV) using cationic liposomes, the antitumor effect of GCV was 47-640 times greater than when the same experiment was performed with a *lacZ* gene. These results demonstrated a potential role of novel cationic liposomes for gene therapy in the treatment of advanced intraperitoneal carcinomatosis [60]. Tumor-associated macrophages play an essential role in tumor growth and metastasis by promoting tumor angiogenesis. To prove this theory, Zeisberger et al. (2006) studied the efficiency of clodronate encapsulated in liposomes (clodrolip) in the murine F9 teratocarcinoma and human A673 rhabdomyosarcoma mouse tumor models; the treatment significantly inhibited tumor growth ranging from 75 to >92% by drastically reducing blood vessel density in the tumor tissue [61]. Further combination of clodrolip with angiogenesis inhibitors shows a promising novel strategy for an indirect cancer therapy. Anti-vascular effects against animal models of lung and ovarian cancer were shown by sterically stabilized immunoliposomes (SIL) loaded with DOX and targeted to the disialoganglioside receptor GD(2) [aGD(2)-SIL(DOX)], which later resulted in selective inhibition of the metastatic growth of experimental models of human neuroblastoma. Chorioallantoic assays depicted that NGR-SL(DOX) substantially reduced the angiogenic potential of various neuroblastoma xenografts, with synergistic inhibition observed for the combination of NGR-SL(DOX) with aGD(2)-SIL(DOX) [62]. To reduce the toxicity for the patients, patients received non-pegylated liposomal DOX in combination with either cyclophosphamide or docetaxel (DTX). The results revealed that the use of non-pegylated liposomal DOX seems to be less toxic than conventional DOX formulations in combination regimens for the first-line therapy of metastatic breast cancer [63]. This led to the hypothesis that arginine-glycine-aspartic acid (RGD) peptide-modified liposomes could increase the efficacy of inhibition of tumor growth by binding with the integrin receptors of tumor cells. To gain evidence for the hypothesis, in vivo studies were performed using a mouse model of drug-resistant MCF7/A. When compared to liposomal DOX alone, the results showed that the sequential treatment of P-glycoprotein (P-gp) gene silencing and cytotoxic drugs with the RGD-modified liposome drug delivery system could be a promising clinical treatment for drug-resistant tumors [64]. Tumor angiogenesis involves multiple signaling pathways that provide potential therapeutic targets to inhibit tumor growth and metastasis. VEGF is known to regulate various signaling pathways in angiogenesis and tumor progression [8]. Recently, VEGF sequence-specific small interfering RNA (siRNA) was used as an anti-angiogenic tumor therapy. Yang et al. (2014) reported that dual-modified liposomes (At-Lp) were designed by attaching two receptor-specific peptides, Angiopep and tLyP-1, which specifically targeted low-density lipoprotein receptor for brain tumor targeting and neuropilin-1 receptor for tumor penetration, respectively [65,66]. Gene transfection and silencing and the antitumor effect of the At-Lp loaded with VEGF siRNA significantly enhanced cellular uptake (2-fold) and down-regulated expression of VEGF in U87 MG glioblastoma cells compared with non-modified and single-modified liposomes. The At-Lp showed great superiority in inhibition of tumor growth, anti-angiogenesis and expression of VEGF and apoptosis effect after in vivo application in nude mice bearing U87 MG glioblastoma and did so without activation of system-associated toxicity and the innate immune response. The authors from this study concluded that the combination of two receptor-specific peptide-mediated liposomes presented a promising platform for effective targeting delivery of siRNA for cancer anti-angiogenic therapy [66]. The VEGF-expression silencing effect was investigated in MCF-7 cells using polycation liposome-encapsulated calcium phosphate NPs (PLCP). VEGF siRNA mediated by PLCP could reduce VEGF expression 60–80%. Furthermore, significant tumor growth and angiogenesis inhibition were observed in a MCF-7 xenograft mouse model when the mice were treated with PLCP/VEGF siRNA or in combination with DOX [67]. There are various methods to upload drugs into liposomes, including adsorption, encapsulation, or emulsion (Figure 6).

### 2.2. Polymeric Nanoparticles (PNPs)

Polymer NPs (PNPs) have elicited much interest in a variety of fields due to their capability to modify the activity of drugs, delay and control the drug release and increase the drug adhesivity or its time of permanence in the skin and they are the platform of choice for nanoparticle-based cancer drug delivery applications [68,69]. PNPs have an average diameter of less than 1 µm; they are named nanocapsules or nanospheres depending on their composition. The preparation of PNPs involves two main strategies: the dispersion of preformed polymers or the polymerization of monomers. PNPs are mainly used in targeted delivery systems as a drug carrier for cancer therapy because of their fundamental characteristic features, including bio-degradability, bio-compatibility, nontoxicity, prolonged circulation and a wide payload spectrum of therapeutic agents [70].

MDR is a serious problem for the treatment of cancer. Understanding the molecular basis of drug resistance in cancer is essential, because such understanding could provide better solutions for more effective treatment of cancer [71]. Novel polymer-lipid hybrid nanoparticle (PLN) formulations were used to deliver the cytotoxic drug, DOX, a chemosensitizer, GG918, or a combination of DOX and GG918. PLNs increase encapsulation and therapeutic efficiency of DOX and GG918 in a human MDR breast cancer cell line (MDA435/LCC6/MDR1) [72].

The cytotoxicity assay exhibited that WOR enhances the efficacy of DOX through an efficient combination delivery accompanied by synergistic drug action. These findings suggest that the use of PNPs increases effective delivery and cytotoxicity with a minimum dose. Similarly, to overcome MDR, dual agent NPs encapsulating the combination of PTX and tariquidar using poly(d,l-lactide-co-glycolide) NPs were prepared and used for simultaneous and targeted delivery of the anticancer drug, PTX, with the P-gp modulator, tariquidar. Initially, NPs were surface functionalized with biotin for active tumor targeting. Under in vitro conditions, dual agents showed significantly higher cytotoxicity in vitro than NPs loaded with PTX alone. In vivo studies also showed significant inhibition of tumor growth. The data from authors indicate that the combination of P-gp modulators and anticancer drugs are a very promising approach to overcome tumor drug resistance [73].

Photodynamic therapy (PDT) is a promising treatment modality for cancer. However, the effectiveness is restricted due to lack of photosensitizers and the generation of lower levels of reactive oxygen species (ROS). To overcome these limitations, OT-alginate NPs were used as a carrier to enhance the therapeutic efficacy through addition of a model photosensitizer, methylene blue. Methylene blue-loaded NPs were evaluated for PDT effectiveness in two cancer cell lines, MCF-7 and 4T1. Encapsulation of methylene blue in NPs significantly enhanced intracellular ROS production and ultimately increased higher cytotoxicity. Therefore, the PNP-mediated modality enhanced its anticancer photodynamic efficacy [74]. Herpes simplex virus thymidine kinase (HSV-TK)-loaded PEG-PBLG NPs are used to treat oral squamous cell carcinoma. PEG-PBLG nanoparticle-mediated HSV-TK/ganciclovir (GCV) showed significant anticancer effect against Tca8113 cells and buccal carcinoma induced in golden hamsters through higher gene-transfer efficiency [75]. Protophorphyrin IX (PpIX, a photosensitizer), -encapsulated chitosan NPs (CNPs) were prepared by self-assembling amphiphilic glycol chitosan-5beta-cholanic acid conjugates in an aqueous environment with an average size of 290 nm. PpIX-CNPs exhibited a sustained release profile in vitro and exhibited enhanced tumor specificity and increased therapeutic efficacy in SCC7 tumor-bearing mice compared to free PpIX. The findings from this study suggest that PpIX-CNPs are a potential anticancer and effective drug delivery system for clinical PDT [76].

A novel PNP-encapsulated curcumin engineered formulation (NanoCurc) shows remarkably higher systemic bio-availability in plasma and tissues compared with free curcumin. Furthermore, xenograft models of human pancreatic cancer revealed significant inhibition of primary tumor growth by the formulation in both subcutaneous and orthotopic settings. The combination of NanoCurc with gemcitabine resulted in even greater inhibition of tumor growth compared to either gemcitabine or NanoCurc alone through significantly reduced expression of nuclear factor-kappa B, matrix metalloproteinase-9 and cyclin D1 [77]. PNPs containing taxanes with an average size of 45 nm show high solubility with enhanced chemoradiotherapeutic efficacy in non-small cell lung cancer by effective internalization into the cells when combined with ionizing radiation (IR) [78]. Administration of autophagy inhibitors such as 3-methyladenine and chloroquine via cholic acid-conjugated NPs of poly (lactic-co-glycolic acid) (PLGA NPs) induces autophagy of the cancer, which is the alternative method for clinical applications [79]. The newly synthesized amphiphilic low molecular-weight heparin LMHA-ATRA conjugate, encapsulating the DOX NPs, exhibited significant loading capacities for DOX with excellent physico-chemical properties, bio-compatibility and remarkable differentiation-inducing activity and anti-angiogenic activity in human breast cancer cells [80]. Recently, NPs engineered with an average size of 50 nm-diameter containing diblock copolymer NPs were used to sequentially release WOR and DTX to cancer cells. The results from the findings suggest that WOR enhanced the therapeutic efficacy of DTX and increased the efficiency of XRT in H460 lung cancer and PC3 prostate cells by releasing the drug in sequential manner without any undesirable toxicity. The results from both in vitro and in vivo studies concluded that DTX/WOR-co-encapsulated NPs are more efficient than each single-drug-loaded NP [81]. Kim et al. (2015) reported the preparation of magnetic nanoparticle-conjugated PMs (MNP-PMs) consisting of PEG-poly(lactide) (PEG-PLA) and iron oxide NPs and used them as nanocarriers for combined hyperthermia therapy and chemotherapy [82]. DOX was encapsulated in MNP-PMs and introduced into hyperthermia temperatures, a condition which is suitable for releasing drugs at a higher rate by using an alternating magnetic field. The combination of MNP-PMs with hyperthermia conditions showed a significant synergistic effect above that of either chemotherapy or hyperthermia treatment alone. Xiong et al. (2015) designed carborane-conjugated amphiphilic copolymer NPs to deliver DOX for the combination of chemotherapy and boron neutron capture therapy, in which DOX-loaded carborane-conjugated PNPs are able to selectively deliver boron atoms and DOX to the tumor site simultaneously in vivo [83]. In order to enhance the efficacy of 17-*N*-allylamino-17-demethoxygeldanamycin (17AAG), an HSP 90 inhibitor, against pancreatic cancer, Rochani et al. (2016) established new nano formulations containing PLGA-coated, 17AAG- and Fe_3_O_4_-loaded magnetic NPs. The aqueous dispensable formulations were able to provide anti-pancreatic cancer activity in the MIA PaCa-2 cell line in a dose- and time-dependent manner in comparison to the murine fibroblast cell lines, L929 [84]. To improve oral bio-availability and efficacy in MDR breast cancer, PLGA NPs were loaded with a chemosensitizer (piperine, PIP) and rapamycin (RPM) formulated for sustained release and effective treatment. This study reveals that the uptake of the RPM (a P-gp substrate) was increased in the presence of the chemosensitizer and also the absorption profile of RPM was increased by using PNPs compared to its suspension counterpart. The findings from this study suggest that the use of a combination of PIP with RPM NPs would be a promising approach in the treatment of breast cancer [85]. Curcumin-loaded chitosan NPs enhance cytotoxicity effect by increasing uptake by cells compared to that of treatment with curcumin alone in cervical cancer [86]. Surnar and Jayakannan (2016) developed a unique biodegradable triple block nanocarrier (TBN) with a hydrophilic PEG outer shell; a middle hydrophobic and biodegradable polycaprolactone (PCL) block for encapsulating anthracycline anticancer drugs like DOX; and an inner carboxylic-functionalized polycaprolactone core for CP drug delivery to enhance the synergistic effect in combination therapy against antagonistic drugs [87]. The developed TBN showed 100% cell growth inhibition in glutathione (GSH)-over-expressed breast cancer cells. The authors demonstrated that although when used alone free drugs were antagonistic to each other, the dual drug-loaded TBN exhibited excellent synergistic cell killing at a much lower drug concentration in breast cancer cells. To enhance the synergistic effects and enhance the therapeutic efficacy, Kumar et al. (2018) developed biodegradable polymeric nanogels with a combination of the histone deacetylase inhibitor, vorinostat and the topoisomerase II inhibitor, etoposide (ETOP) [88]. The resulting product showed sustained release and enhanced synergistic effect on human cervical HeLa cancer cells via caspase 3/7 activation. Therefore, combination of polymeric NPs with anticancer drugs could provide a promising strategy for cancer therapy with enhanced anticancer effect.

### 2.3. Polymeric Micelles (PMs)

PMs are nanoscopic core/shell structures formed by self-assembly amphiphilic block copolymers or graft copolymers with an average diameter between 10 and 100 nm and are spherical in shape. PMs have high thermodynamic and kinetic properties and have been used in site-specific delivery of anticancer drugs to tumors [89]. Bae et al. (2007) reported using a conjugation of poly(ethylene glycol)-poly(aspartate hydrazide) with DOX and a phosphatidylinositol-3 kinase inhibitor wortmannin (WOR) with unimodal micelle structure with a <100 nm particle size [90]. Composite micelles containing both cisplatin (CP) (IV) prodrug and PTX could release effective anticancer drug CP (II) in the cancer cells upon cellular reduction and PTX via acid hydrolysis. The composite micelles displayed a synergistic effect with reduced systematic toxicity and enhanced antitumor efficacy [91]. To increase the efficiency of synergistic tumor therapy, Zheng et al. (2013) developed cationic micelles using the triblock copolymer poly(ethylene glycol)-b-poly(l-lysine)-b-poly(l-leucine) (PEG-PLL-PLLeu) to systemically co-deliver DTX and siRNA-Bcl-2 for an effective drug/gene vector. The results showed that the micelle complexes with siRNA-Bcl-2 effectively knocked down the expression of Bcl-2 mRNA and protein, led to downregulation of the anti-apoptotic Bcl-2 gene and enhanced antitumor activity with a smaller dose of DTX. These methods proved that cationic micelles significantly inhibited tumor growth in the MCF-7 xenograft murine model as compared to the individual siRNA- and DTX-only treatments [92]. To increase the therapeutic index of cancer chemotherapy, Wang et al. (2014) developed a combination of landscape phage and micellar NPs. The combination of PTX with the self-assembled nanopreparation composed of MCF-7-specific phage protein and PEG-PE micelles showed selective toxicity to target cancer cells but not to non-target, non-cancer cells [93]. Both in vitro and in vivo various cellular and apoptotic assays showed extensive tumor reduction and extensive necrosis as a result of improved tumor delivery of PTX. Hence, micellar-mediated combined therapy could provide support as an effective and safe chemotherapy for improved breast cancer treatment [93]. Generally, the PNP therapy is applicable for controlled release of multiple chemotherapeutic drugs. Based on this concept, a dual-drug co-delivery system was developed using a nanocarrier called amphiphilic diblock copolymer, PCL-b-P (OEGMA-co-AzPMA) with DOX and platinum (IV) (Pt[IV]). The results suggested that with the developed dual-drug co-delivery system, micelles were effectively taken up by the cells and were able to simultaneously release drugs into the cells [94].

PMs are formed by spontaneous assembly from amphiphilic polymers in selective solvents to decrease the free energy. Micelles have several benefits such as their spherical shell–core structure, their small size and their capacity to be sterilized by simple filtration [95]. Taillefer et al. (2000) prepared pH-responsive PMs consisting of random copolymers of N-isopropylacrylamide, methacrylic acid and octadecyl acrylate [96]. The prepared micelles were successfully loaded with a substantial amount of a photoactive anticancer drug, namely, aluminum chloride phthalocyanine (AlClPc). PH-responsive PMs loaded with AlClPc were found to exhibit higher cytotoxicity against EMT-6 mouse mammary cancer cells in vitro than the control Cremophor EL formulation. PMs are frequently used to solubilize poorly soluble PDT agents such as meso-tetratphenylporphine (TPP). For instance, encapsulation of TPP into PEG-PE-based micelles and immunomicelles significantly increased the anticancer effects of PDT against murine (LLC, B16) and human cancer cell lines (MCF-7, BT20) respectively [97]. Similarly, the authors studied TPP-loaded PEG-PE micelles using a tumor-specific monoclonal 2C5 antibody, which resulted in a significantly improved anticancer effect of the drug under the PDT conditions against murine LLC cells in female C57BL/6 mice [98]. Iyer et al. (2007) developed PMs of zinc protoporphyrin (ZnPP) with a water-soluble, bio-compatible and amphiphilic polymer, PEG, to target tumor tissues selectively based on the EPR effect [99]. The complex micellar formulations, SMA-ZnPP and PEG-ZnPP, increased tumor-cell killing by generating reactive singlet oxygen. The findings from this study suggest that these water-soluble PMs of ZnPP are potential candidates for targeted chemotherapy as well as PDT. PEG-block-PLA micelles were used to deliver multiple poorly water-soluble drugs including PTX, ETOP, DTX and 17-AAG at clinically relevant doses [100]. The combination of anticancer agents with bevacizumab, an anti-vascular endothelial growth factor humanized monoclonal antibody and 7-ethyl-10-hydroxycamptothecin (SN-38) incorporated into a polymeric micelle exhibited significant anticancer activity against human lung cancer cells [101]. To enhance the efficacy of combined modality anticancer therapy, PMs were developed using *N*-(2-hydroxypropyl) methacrylamide (HPMA), which is known to improve the retention of intratumorally administered chemotherapeutic agents at the pathological site and to raise the therapeutic index. In vivo data suggest that HPMA increases a synergistic interaction between XRT and chemotherapy [102]. Simultaneous delivery of an anticancer drug and siRNA by a conjugation of deoxycholic acid (DA3) and a low molecular weight polyethylenimine (PEI 1.8 kDa) significantly increased gene silencing efficiency above that of PEI 25 kDa alone, even in the presence of endocytosis inhibitors, suggesting that the polymeric carrier can mediate an endocytosis-independent macromolecular transduction similar to that of cell-penetrating peptides. The micelle-like core-shell structure enables the conjugates to encapsulate and dissolve PTX and it can interact with siRNA to form stable complexes (PTX/DA3/siRNA). Administration of PTX/DA3/siRNA into the cells as well as to tumor-bearing animals showed significant inhibition of cancer cell growth [103]. A study was performed to evaluate the effect of newly formulated micelles containing an amphiphilic graft copolymer, *N*-octyl-*O*-sulfate chitosan and its PTX-encapsulated micelles (PTX-M) in human HepG2 cells and the MDR mutant, HepG2-P. The complex micelles showed effective inhibition through activation of various mechanisms that stimulated P-gp ATPase, competitively impeding the binding of PTX with P-gp and reducing the fluidity of the cell membrane. As result, PTX-M presented the highest cellular uptake and the lowest efflux rate of PTX reported in mouse tumor models [104]. Salzano et al. (2015) developed self-assembly PMs that are able to efficiently co-load an anti-survivin siRNA and PXL (survivin siRNA/PXL PM) in PXL-resistant ovarian cancer [105]. The results suggest that in mice xenografted with SKOV3-tr there was significant downregulation of survivin expression in tumor tissues together with a potent anticancer activity by the complex micelles. Amphiphilic polycarbonate/PEG copolymer with a star-like architecture was used for efficient delivery of anticancer drugs. For instance, DOX-loaded NPs self-assembled from the star-like copolymer exhibited greater kinetic stability and higher DOX-loading capacity than micelles prepared from a cholesterol-initiated diblock analogue. DOX-loaded vesicles injected into 4T1 tumor-bearing mice exhibited enhanced accumulation in tumor tissue due to the EPR effect. Importantly, DOX-loaded vesicles demonstrated greater tumor growth inhibition than free DOX without causing significant body weight loss or cardiotoxicity [106]. Kulhari et al. (2015) reported the synthesis of cRGDfK peptide-conjugated succinoyl-tocopheryl polyethylene glycol succinate nanomicelles for targeted delivery of DTX. cRGDfK peptide could specifically target αvβ3 integrin receptors, which are mostly overexpressed in tumor cells only [107]. Peptide-conjugated DTX-loaded nanomicelles displayed high anti-angiogenic effect compared to unconjugated nanomicelles in DU145 human prostate cancer cells and human umbilical vein endothelial cells. Biodegradable PMs with co-encapsulated PTX and honokiol showed a significant anticancer effect against breast cancer cells through sustained release of drug, increased cellular uptake and cytotoxicity, both in vitro and in vivo. The findings from this study indicated that drug-loaded PMs achieve greater levels in tumor tissues compared to the free drug [108]. Ruttala et al. (2017) developed pH- and redox-responsive crosslinked polypeptide-based micelles for enhanced chemotherapeutic efficacy and minimized side effects through increased stability and drug release properties [109]. The crosslinked micelles, carrying DTX and lonidamine (LND), exhibited a significantly greater anticancer effect compared to that of non-crosslinked micelles; this method ultimately increased the intracellular ROS level and oxidative stress and caused damage to intracellular components that resulted in greater apoptosis of cancer cells than when either DTX or LND was used alone. Therefore, the combination of PMs and anticancer drugs exhibited potential increased inhibition of tumor growth without any undesired side effects, thereby enhancing the safety profile.

### 2.4. Dendrimers

Dendrimers are highly defined artificial macromolecules, which are nano-sized, radially symmetric with well-defined, homogeneous and monodispersed structure consisting of tree-like arms or branches; they have a combination of many functional groups and a compact molecular structure [110,111]. PEG-dendrimer, H(2)N-PEG-dendrimer-(COOH)(4) was used as a carrier for a combination of PTX and alendronate (ALN). The combination of PTX-PEG-ALN within the conjugate exhibited an improved pharmacokinetic profile compared with the free drugs without any solubilizing agent for cancer bone metastases [112]. Dendritic polymers analogous to proteins, enzymes and viruses are easily functionalized and have several advantages, including providing long life to drugs conjugated with dendrimers, high stability, water solubility and decreased immunogenicity and antigenicity. Having all these characteristics, dendrimers have gained interest for use in several biomedical applications including drug delivery, gene delivery, magnetic resonance imaging contrast agents and sensors [111,113,114]. In addition, these dendrimers used as carrier molecules could provide improved target specificity achieved by passive and/or active targeting mechanisms. To improve the efficacy and reduce the side effects of anticancer drugs, pH-activated polymers were used as a drug-delivery vehicle system with photochemical internalization (PCI) capability, which is specifically used for site-specific delivery of membrane impermeable macromolecules from endocytic vesicles into the cytosol. To prove this phenomenon, DOX was conjugated to polyamidoamine (PAMAM) dendrimers via pH-sensitive and -insensitive linkers and was combined with different PCI providers. The resultant product showed that PCI strategies significantly improved the cytotoxicity of free DOX on Ca9-22 cells at higher concentrations. The “light after” PCI treatment was efficient in releasing DOX from the PAMAM-hyd-DOX conjugates, resulting in more nuclear accumulation of DOX and more cell death through synergistic effects [115]. Gardikis et al. (2010) demonstrated that the loading of DOX in liposomal locked-in dendrimers enhances drug encapsulation and a slower drug release rate more efficiently compared to pure liposomes [116]. PEG-dendrimer, H(2)N-PEG-dendrimer-(COOH)(4), being used as a carrier for a combination of PTX and ALN. The combination of a PTX-PEG-ALN conjugate was designed to exploit active targeting by the ALN molecule and passive targeting through the EPR effect. The resultant complex conjugate, PTX-PEG-ALN, exhibited an improved pharmacokinetic profile compared with the free drugs, based on the marked increase in their half-lives [80]. Thomas et al. (2013) designed multivalent dendrimers known as methotrexates as a folate-targeting anticancer therapeutic agent to serve as both a cancer cell-specific targeting ligand and as a therapeutic cytotoxic agent [117]. The resultant new methotrexate-conjugated PAMAM dendrimers, each carrying multiple copies of methotrexate attached through a stable amide linker, exhibited potent dual activity. The phosphatidylinositol 3-kinase/Akt pathway plays a significant role in cell survival and apoptosis and also has been shown to display a critical involvement in ovarian cancer, making it an attractive therapeutic agent target. Therefore, Kala et al. (2014) designed a combination of a triethanolamine-core PAMAM dendrimer which forms stable NPs with the Akt siRNA, can protect the siRNA against RNase digestion and is highly effective for initiating Akt target-gene silencing both in vitro and in vivo; administration of dendrimer-nanovector-mediated siRNA delivery to target Akt, combined with PTX to target cancer cells, shows potentially effective and potent anticancer activity in ovarian cancer [118]. Multifunctional theranostic platforms capable of concurrent near-infrared (NIR) fluorescence imaging and phototherapies are strongly recommended for cancer diagnosis and treatment. Dendrimer-encapsulated naphthalocyanine as a single agent-based theranostic nanoplatform was used as an NIR fluorescence imaging and combinatorial anticancer phototherapeutic molecule. An example of such a molecule is SiNc encapsulated into the hydrophobic interior of a generation 5 polypropylenimine dendrimer by surface modification with PEG. In vitro and in vivo studies confirmed that phototherapy mediated by SiNc efficiently destroyed chemotherapy-resistant ovarian cancer cells. Solid tumors treated with a single dose of SiNc-NP combined with NIR irradiation were completely eradicated without cancer recurrence [119]. Multicomponent 5-fluorouracil (5-FU)-loaded PAMAM, stabilized-silver (Ag) nanocomposites were developed to enhance apoptosis in human lung cancer cells. The generated nanocomposites showed sustained release of 5-FU from nanocomposites and exhibited a synergistic anti-proliferative effect in A549 (human lung cancer) and MCF-7 (human breast cancer) cells by increasing the levels of ROS [120]. Kuruvilla et al. (2017) developed dendrimer-DOX conjugates to improve the therapeutic index of DOX delivered via HAI by loading the drug onto generation 5 PAMAM dendrimers targeted to hepatic cancer cells (HCC) via N-acetylgalactosamine (NAcGal) ligands [121]. The intratumoral delivery of free DOX into HCC-bearing nod SCID gamma mice exhibited a 2.5-fold increased level of inhibition of tumor growth compared to controls. P1 and P2 particles combined with NAcGal-targeting with L3- or L4-DRX linkages increased inhibition to 5-fold that of controls and also increased the therapeutic index of DOX.

### 2.5. Microspheres

The therapeutic efficiency of combined chemotherapy and gene therapy was evaluated on the human hepatocellular carcinoma cell line, HepG2, using double-walled microspheres that consisted of a poly (d,l-lactic-co-glycolic acid) (PLGA) core surrounded by a poly(l-lactic acid) shell layer to deliver DOX and/or chitosan-DNA NPs for delivering the gene encoding the p53 tumor suppressor protein (chi-p53) to the HepG2 cells. The results depicted that the combined DOX and chi-p53 treatment exhibited enhanced cytotoxicity compared to either DOX or chi-p53 treatment alone. Collectively, the authors presented double-walled microspheres as a promising dual anticancer delivery system for combined chemotherapy and gene therapy [122].

### 2.6. Carbon Nanoparticles and Carbon Based Nanosystems

Carbon-based nanomaterials such as graphite (GT), ND, fullerenes, CNTs, GO, reduced GO (rGO) and GO-Ag NP nanocomposites have been widely used in biomedical applications such as diagnostic and therapeutic purposes due their versatile properties such as surface-to-volume ratio, thermal conductivity, rigid structural properties capable of post-chemical modification and excellent bio-compatibility. A majority of the graphene-based materials used for antibacterial and anticancer therapy are drawn from GT, (the precursor materials for graphene), GO and rGO and GO-Ag nanocomposites [123].

#### 2.6.1. Nano Diamond

ND is an allotrope of carbon with an average particle size between 4 and 6 nm and forms aggregates in the range of 100–200 nm [124]. ND is frequently used for enhancing water solubility for anticancer drugs, including Purvalanol A and 4-hydroxytamoxifan, which are known as potent molecules for liver and breast cancers [125]. DOX incorporated into negatively charged NDs in combination with NaCl and cationic DOX ions enhances the therapeutic efficiency and loading capacity and therefore the cytotoxicity in the HT-29 colorectal cancer cell line. To increase cancer-targeting properties, 10-hydroycamptothecin was loaded into the clustered ND [126] and an epirubicin-loaded ND complex was made with anti-EGFR antibodies using lipids to make a thin film on the surface of the ND [127]. DOX-loaded NDs increased the reduction of myelosuppression in 4T1 murine mammary tumors. All these studies suggest that NDs could be safer nanocarriers for long-term DOX treatment [128]. Zhang et al. developed fluorescein-labeled dT-nucleotides for PTX gene loading and an anti-EGFR monoclonal antibody for selective targeting of PTX on the surface of NDs with the hetero-bifunctional crosslinker sulfosuccinimidyl 6-(3’-[2-pyridyldithio]-propionamido) hexanoate [129]. The developed complex molecule, PTX-DNA/mAb@ND, selectively entered into EGFR-overexpressing MDA-MB-231 cancerous cells compared to basal EGFR-expressing MCF7 cells.

CP-loaded ND shows reduced cytotoxic effects by delivering low concentrations of CDDP in the blood and a higher concentration of drug in the acidic cytoplasm, which suggests that the complexation between CDDP and the carboxyl groups on the ND surface behaves in a pH-dependent manner in human cervical cancer cells [130]. NDs with an average size of 3.5 nm were conjugated to PTX by chemical modifications. Treatment with 0.1–50.0 µg/mL ND-PTX, after 24 h exposure to these composites, significantly reduced the cell viability in A549 human lung carcinoma cells by arresting mitotic cell division and inducing apoptosis in the cells in a concentration-dependent manner. Furthermore, ND-PTX markedly blocked the tumor growth and formation of lung cancer cells in a xenograft SCID mice model [131]. Alhaddad et al. (2011) synthesized a fluorescent ND vector with two different cationic coatings, for siRNA delivery into Ewing sarcoma cells (EWS) in culture [132]. This vector showed greater transfection efficiency than the conventional transfection agent called Lipofectamine by inhibiting the EWS-Fli1 expression, combined with a lower toxicity to the cells in serum-supplemented medium. The newly designed vector could promote bio-distribution and less toxicity to the cells. Because tumor-bearing mice treated with ND-DOX complex molecules exhibited significantly enhanced life spans compared to mice treated with conventional free DOX, this study concluded that ND-DOX could be a promising nanodrug with potential chemotherapeutic efficacy and safety [133]. To enhance the intracellular uptake and eliminate the non-specific binding of anticancer drugs into cancer cells, ND that carried PTX combined with cetuximab (Cet), a specific monoclonal antibody against epidermal growth factor receptor (EGFR), was designed. The ND co-delivery of PTX and Cet induced mitotic catastrophe and tumor inhibition in human colorectal cancer (CRC). Surprisingly, ND-PTX-Cet significantly decreased tumor size in the xenograft model of EGFR-expressing human CRC tumors in nude mice. This study suggests that the co-delivery of PTX and Cet by ND enhanced the effects of mitotic catastrophe and apoptosis in vitro and in vivo [134].

Recently, two allotropes of carbon, such as the nanotube and the fullerene, gained much interest for use in biological applications. Oberdorster et al. (2004) demonstrated that the toxic effect of water-solubilized C60 fullerenes was not observed in rodents when underivatized C60 fullerenes were used [135,136]. Nanocrytalline (C60) exhibited significant anticancer effects against the rat glioma cell line C6 and the human glioma cell line U251 by regulating multiple pathways in a dose-dependent manner. At high concentration (1 μg/mL) it induces ROS-mediated necrotic cell damage that was partly dependent on oxidative stress-induced activation of extracellular signal-regulated kinase (ERK), whereas at low concentration (0.25 μg/mL) it did not induce either necrotic or apoptotic cell death but caused oxidative stress/ERK-independent cell cycle block in G(2)/M phase and subsequent inhibition of tumor cell proliferation [137].

Zogovic et al. (2009) also demonstrated that nanoC(60) induced cell death in mouse B16 melanoma cells by oxidative stress, mitochondrial depolarization and caspase activation, leading to apoptotic and necrotic death [138]. Intraperitoneal administration of nanoC(60) into tumor-bearing mice for two weeks significantly augmented tumor killing by a significant increase in splenocyte production of the immunoregulatory free radical nitric oxide. The cellular internalization of buckysomes embedded with the hydrophobic fluorescent dye 1,1′-dioctadecyl-3,3,3′,3′-tetramethylindocarbocyanine perchlorate embedded with PTX reduced the viability of MCF-7 breast cancer cells compared to that of Abraxane, a conventional treatment drug for breast cancer. Interestingly, the buckysomes were not cytotoxic. The results of these studies suggest that buckysomes prepared from self-assembly of AF-1 at 70 °C are promising nanomaterials for the delivery of hydrophobic molecules [139]. To improve fullerene-specific properties, a well-defined fullerene-DOX conjugate was developed. The resulting conjugate was distributed mostly in the cytoplasm in contrast to free DOX molecules [131]. C60 fullerenes use high antioxidant activity and the blocking of specific cell receptors, such as EGFR, to efficiently inhibit the growth of transplanted malignant tumors [140]. Human connective tissue-derived fibrosarcoma cells, HT1080, exposed to 3-h administration of PEG-fullerene [C60] experience cell death via intracellular DNA fragmentation through the mechanism of ROS such as hydroperoxides and peroxyl radicals, or superoxide anion radicals [141]. Sonodynamically-induced antitumor effects of polyhydroxy fullerenes (PHF) was evaluated in sarcoma 180 cells and solid tumors from colon 26 carcinoma cells. Sonication induced cell death, which was enhanced by two-fold in the presence of 80 μM PHF. The combined treatment of ultrasonic exposure with PHF suppressed the growth of implanted colon 26 tumors by oxidative stress and lipid peroxidation [142]. Furthermore, the authors demonstrated that in HL-60 cells subjected to 2-MHz continuous ultrasound in the presence of PHF for 3 min, the number of apoptotic cells after sonodynamic exposure was significantly higher than after single treatments, such as ultrasound alone or PHF alone [143]. DOX (total dose 2.5 mg/kg) administered in combination with C60 fullerene (total dose 25 mg/kg) in tumor-bearing animals resulted in tumor growth inhibition, prolonged life, metastasis inhibition and increased apoptosis among tumor cells and was more effective than DOX alone [143].

#### 2.6.2. CNT

CNTs are sp2-hybridized carbon materials formed by rolling up hexagonal graphene sheets. There are two types of CNTs: single walled CNTs (SWCNTs) and multiwalled CNTs (MWCNTs). Due to the unique chemical and physical properties of CNTs, they have been used in a wide variety of applications. However, for usage in biological systems, solubility is a critical factor; therefore, CNTs require organic molecules to enhance their solubility. For example, acid treatment to generate carboxylic end groups and incorporation of polymers are representative examples of measures to improve CNT solubility [144]. Ajima et al. (2005) demonstrated that oxidized single-wall carbon nanohorns (SWNHs) can serve as drug delivery agents [145]. To develop SWNHs as carrier agents, initially CP was entrapped into them; the results showed that the CP structure was maintained inside the SWNHs and that the CP was released slowly and at a steady state in aqueous environments. However, the released CP was more effective to inhibit the growth of human lung-cancer cells, while the SWNHs themselves had no such effect.

To enhance biological delivery applications and efficient gene therapeutic purposes, Jia et al. (2007) prepared a novel double functionalization of CNTs containing antisense oligodeoxynucleotides (ASODNs), a therapeutic gene and quantum dot labeling (CdTe-tagged ASODNs-f-MWCNTs) via electrostatically layer-by-layer assembly [146]. The resultant product showed efficient intracellular transporting, strong nuclear localization and high delivery efficiency of ASODNs by the PEIMWCNTs carriers. The supramolecular complexes of MWCNTs and DOX were prepared as anticancer agents using block copolymers to bind with both the aromatic chromophore and DOX via π-π stacking. The resultant complex showed significant cytotoxicity activity in MCF7 human breast cancer cells [147]. To specifically target squamous cancer cells, epidermal growth factors (EGF) attached to SWCNTs were combined with quantum dot luminescence (SWCNT-Qdot-EGF bio-conjugates); these conjugates were internalized rapidly into the cancer cells of head and neck squamous carcinoma cells (HNSCC) overexpressing EGFR. SWCNT-Qdot-EGF conjugates injected into live mice were selectively taken up by HNSCC tumors but SWCNT-Qdot controls with no EGF were cleared from the tumor region in <20 min. HNSCC cells treated with SWNT-CP-EGF also exhibited efficient anticancer effects compared to control treatment. Most significantly, regression of tumor growth was rapid in mice treated with targeted SWCNT-CP-EGF relative to non-targeting SWCNT-CP [148]. Most platinum-based drugs have short circulation times in the blood, which leads to reduced intracellular and DNA binding. To overcome these limitations, an SWCNT-mediated Pt(IV) prodrug delivery complex was synthesized using a folate derivative at an axial position of the CNT, which could target over-expressed folate receptor (FR). The resultant SWCNTs deliver the folate-bearing Pt(IV) cargos into FR(+) cancer cells by endocytosis [149]. DOX was used to target tumors by using folic acid (FA), a targeting agent for many tumors and it was released specifically into the lysosomes in a controlled manner by using single wall SWCNTs. The released DOX causes significant damage to nuclear DNA and inhibits proliferation in HeLa cells [150]. To overcome MDR in cancer therapy, water-soluble SWCNTs loaded with DOX and the antibody to P-gp (AP), were synthesized to form DOX/Ap-SWCNTs and administered to MDR human leukemia cells (K562R). The result suggest that the synthesized complex showed effective loading capacity and controlled release of DOX in the K562R cells following exposing to NIR. Ap-SWNTs loaded with drug molecules can be used to suppress the proliferation of MDR cells, to destroy the tumor stem cells and to inhibit the tumor metastases. Carboplatin 0.20 mg-loaded CNTs and carboplatin 0.13 mg-loaded carbon nanofibers (CNFs) exhibited cytotoxicity in urological tumor cell lines, which was dependent on the drug release. Both CNTs and CNFs loaded with CP seemed to be effective, though CNT-CP was more effective than CNF-CP proliferation impairment and clonogenic survival of tumor cells [151]. In order to enhance the solubility, initially MWCNTs) were first modified to include hyperbranched poly citric acid (PCA) and then, PTX was conjugated to the carboxyl functional groups of PCA via a cleavable ester bond to obtain a MWCNT-g-PCA-PTX conjugate. When administered into tumor tissues and tumor cells, the conjugate’s release of PTX was faster at pH 6.8 and 5.0 than at 7.4. Furthermore, the in vitro toxicity effect was significant in human lung (A549) and ovarian cancer cell lines (SKOV3) [152]. The photothermal effect of SWCNTs was performed in combination with the anticancer drug DOX for selective killing of breast cancer cells with PEG bio-functionalized and DOX-loaded SWCNTs conjugated with FA. The conjugate released DOX effectively in the tumor environment. Due to strong optical absorbance of SWCNTs in the NIR region, SWCNTs exhibited strong light-heat transfer characteristics. The combination of laser and DOX-conjugated SWCNTs displayed enhanced and accelerated killing of breast cancer cells [153]. The efficiency of SWCNTs conjugated with antibody C225, which could bind specifically with over-expressed EGFR in colorectal cancer cells and a chemotherapeutic drug called 7-Ethyl-10-hydroxy-camptothecin (SN38) was evaluated in three different colorectal cancer cell lines: HCT116, HT29 and SW620. The findings suggest that the cellular uptakes of SWCNT in EGFR-over-expressing cells (HCT116 and HT29) were much higher than that of the negative control (SW620), measured by receptor-mediated endocytosis [154]. Cao et al. (2015) developed a new carrier system for encapsulation of DOX for targeted delivery to cancer cells over-expressing CD44 receptors using PEI-modified MWCNTs [155]. The modified MWCNTs conjugated with fluorescein isothiocyanate (FI) and hyaluronic acid (HA). The newly formed MWCNT/PEI-FI-HA/DOX complexes exhibited increased drug loading capacity, up to 72% and the complexes are water soluble and stable. The results suggested that the drug release rate was higher in acidic conditions (pH 5.8, tumor cell microenvironment) than in physiological conditions. The developed HA-modified MWCNTs hold great promise for use as an efficient anticancer drug carrier for tumor-targeted chemotherapy. To gain the evidence to prove that surface coating is essential to enhance the cytotoxicity, biopolymer-coated SWCNT was functionalized with the anticancer drug, betulinic acid, in the presence of Tween 20, Tween 80, PEG and chitosan. The findings showed that all the chemically coated samples seemed to release the drug in a controlled and sustained manner compared to the uncoated ones. Further cytotoxicity effects of various coated SWCNTs in mouse embryonic fibroblast cells (3T3) revealed that the cytotoxicity was dependent upon the drug release profiles as well as the chemical components of the surface-coating agents [156]. SWCNTs-DOX increased the rate of cell death of MDA-MB-231 cells in the presence of NIR compared to DOX and PTT alone, by accumulation of SWCNTs-DOX inside the cells at high concentration; this complex effectively localizes into the MDA-MB-231 cell nucleus and eventually induces cell death by mitochondrial disruption and ROS generation. Targeting activation of apoptosis in cancer cells is an effective strategy for cancer therapy. To overcome current limitations, Kim et al. (2017) developed PEG-coated CNT-ABT737, a nanodrug that improved the mitochondrial targeting in lung cancer cells by abruption of the mitochondrial membrane potential [157]. The complex internalized into the early endosomes via macropinocytosis and clathrin-mediated endocytosis and was eventually delivered into the mitochondria.

#### 2.6.3. Graphene Oxide

GO was extensively used in a wide variety of applications including antibacterial and anticancer agents. The nanoscale GO (NGO) is functionalized with sulfonic acid groups, followed by covalent binding of FA molecules to the NGO. Chemotherapeutic agents such as DOX and camptothecin (CPT) are conjugated with NGO via π-π stacking. Administering FA-NGO-conjugated chemotherapeutic agents into MCF-7 cells displayed improved therapeutic efficacy compared to that of a single drug. The RNAi technique is an effective method to inhibit protein expression by targeted cleavage of mRNA specifically in MDR cancer cells [158]. Sequential delivery of Bcl-2-targeted siRNA and the anticancer drug DOX into HeLa cells was done using PEI-functionalized GO. The findings from this study reported a synergistic effect, which leads to a significantly enhanced chemotherapy efficacy [159]. The MWCNTs and GO functionalized by highly hydrophilic and bio-compatible poly (vinyl alcohol) used for loading and delivery of CPT. The synthesized complex efficiently induced cell death in human breast and skin cancer cells [160]. The chitosan-functionalized GO (CS-GO) shows potential loading capacity. CPT and GO-CS-CPT complexes show remarkably high cytotoxicity in HepG2 and HeLa cell lines compared to the pure drug and also are used for efficient gene transfer [161]. The combination of chemo and photothermal therapy (PTT) seems to be interesting in cancer therapy using polyvinylpyrrolidone- functionalized NGO. FA is an ideal pH-responsive nanocarrier for delivery of the anticancer drug DOX with a loading ratio of more than 100%. The results from in vitro experiments with the combination of chemotherapy and NIR PTT demonstrated that the targeted chemo-PTT could specifically deliver drug and heat to tumor sites and showed excellent efficacy as an anticancer therapy [162]. Human multiple myeloma cells treated with GO and GO loaded with DOX significantly inhibited cell proliferation compared with either DOX or GO alone [163]. rGO nanosheets coated with an anti-angiogenic anticancer taurocholate derivative of low-molecular-weight heparin (LHT7) provided greater loading capacity for DOX compared to uncoated rGO nanosheets. Following intravenous administration into KB tumor-bearing mice, in vivo tumor accumulation of LHT-rGO/DOX was 7-fold higher than that of rGO/DOX 24 h post-dosing [164]. Zheng et al. (2016) developed highly efficient bio-functionalized rGO for nuclear delivery of anticancer drugs using anti-HER2 antibody-conjugated poly-l-lysine functionalized rGO (anti-HER2-rGO-PLL) [165]. The prepared nanocarriers, with anti-HER2 serving as a guide, efficiently delivered DOX to the nucleus in HER2-over-expressing cancer cells. Cellular uptake of anti-HER2-rGO-PLL into MCF7/HER2 cells is significantly higher than that of rGO-PLL alone. The anti-HER2-rGO-PLL/DOX potentially promoted accumulation of DOX inside the nucleus, which in turn led to an increase in anticancer efficacy compared to rGO-PLL/DOX. In another study, the synergistic effect of using multiple treatment drugs was determined in different types of cancer cells such as MDA-MB-231, MCF-7 and BT474 cells by a polyethylene glycosylated (PEGylated) lipid bilayer-wrapped NGO co-loaded with DOX and rapamycin (RAPA), referred to as GOLDR. Treatment with the free drugs in combination (DOX and RAPA) presented a synergistic anticancer effect in MDA-MB-231, MCF-7 and BT474 cells. The treatment with GOLDR and NIR laser irradiation-induced photothermal effects of NGO resulted in higher chromatin condensation and apoptotic body formation and enhanced protein expression of apoptosis-related markers (Bax, p53, p21 and c-caspase 3). This type of approach provides effective chemo-PTT for resistant cancers [166].

A large surface area with abundant functional groups makes GO an attractive platform as an anchoring site for various NPs such as platinum (Pt), Ag, TiO_2_, Fe_2_O_3_, CeO_2_ and PbS to form GO-based nanocomposites [167]. The reduction of GO and its nanocomposites has been used to fabricate a variety of rGO/NP hybrid materials for a wide range of potential applications requiring properties such as optical, electronic, thermal and mechanical [167] and also various biological applications such as antibacterial and anticancer [168,169,170]. Shen et al. (2012) prepared a multifunctional nanocomposite GO-PEG-FA/gadolinium(Gd)/DOX-containing GO conjugated with PEG, modified by FA and containing a magnetic resonance imaging (MRI) probe, gadolinium-diethylenetriamine-pentaacetic acid-poly(diallyl dimethylammonium) chloride (Gd-DTPA-PDDA) [171]. MRI testing revealed that GO-PEG-FA/Gd/DOX exhibited superior efficiency of imaging the targeted tumor over free Gd-(3+). The in vitro release of DOX from the nanocomposite under tumor-relevant conditions (pH 5.5) was fast during the initial 10 h and then become relatively slow. The authors experimentally demonstrated that the multifunctional nanocomposite exhibited a cytotoxic effect upon cancer cells, suggesting that this nanocomposite could be used as a potential candidate to detect and specifically treat early malignancies. To improve the stability of nanoscale GO and efficient loading of DOX, a nanohybrid NGO of dextran NGO-HDex with DOX was formed. The cell viability assay indicated that the NGO-HDex displayed lower cytotoxicity against MCF-7/ADR cells than did the native NGO. DOX-loaded NGO-HDex displayed a more efficient killing effect in the cells than did free DOX. Therefore, NGO-HDex is a potential anticancer agent for killing drug-resistant cancer [172]. To address controlled release of drugs, Wang et al. (2014) designed pH-sensitive konjac glucomannan/sodium alginate (KGM/SA) and KGM/SA/GO hydrogels using GO as a drug-binding effector for anticancer drug loading and release. The findings from this study revealed that GO significantly enhanced the release of 5-FU from 38.02% at pH 1.2 to 84.19% at pH 6.8 after 6 h and 12 h, respectively. Therefore, the KGM/SA/GO-3 composite seems to be suitable as an effector molecule for drug delivery of 5-FU [173].

A multifunctional graphene-based nanohybrid, GN/Fe_3_O_4_/PF127 was prepared using a simple and facile “one-pot” process consisting of simultaneous reduction of GO/Fe_3_O_4_ and subsequent assembly of Pluronic F127 (PF127) onto graphene nanosheets; this nanohybrid acted as a better platform for chemophototherapy when combined with the therapeutic DOX in HeLa cells. The hybrid contains a unique grouping of components: an MRI contrast agent (Fe_3_O_4_ NPs), DOX and a soluble facilitator (PF127), which provides physiological dispersity and stability to the nanohybrid. The GN/Fe_3_O_4_/PF127 nanohybrid exhibits a photothermal and cytotoxic effect [174]. Combination therapy utilizing PTT and chemotherapy seems to be a potential therapeutic strategy for cancer. A nanocomposite was prepared by the addition of CuS NPs on GO functionalized with PEG (PEG-GO/CuS). The prepared nanocomposite exhibited significant bio-compatibility, high storage capacity for anticancer drugs such as DOX and high photothermal conversion efficiency. DOX-loaded PEG-GO/CuS nanocomposites (PEG-GO/CuS/DOX) displayed significant cytotoxicity both in vitro and in vivo in a cervical cancer model system. The results from this study indicated that single application of either chemotherapy or PTT seems to be less significant than combined treatment [175]. Similarly, Pt(IV)-conjugated NGOs show potential for targeted drug delivery when combined with photothermal-chemotherapeutic agents under NIR laser irradiation [176]. Gurunathan et al. (2015) performed comparative studies to determine the efficiency of various nanomaterials such as GO, rGO, Ag NPs and rGO-Ag NPs in ovarian cancer [168]. Among the tested nanomaterials, rGO-Ag NPs showed potential cytotoxicity against human ovarian cancer cells at low concentration. Therefore, Ag-decorated rGO nanocomposites show potential as more potent anticancer agents than other materials, because they offer two different agents in a single platform. Similarly, Khan et al. (2016) demonstrated that NPs with Ag-decorated rGO show more potent cytotoxicity against human lung cancer cells than conventional anti-cancer drugs [177]. A synergistically enhanced tumoricidal effect was observed in hyperthermia conditions by treatment with a multifunctional nanocomposite consisting of GO-iron oxide-DOX (GO-IO-DOX), which provided pH-dependent drug release and enhanced T2 contrast for MRI [178]. Lactobionic acid and carboxymethyl chitosan-functionalized GO nanocomposites exhibited significant bio-compatibility in the liver cancer cell line SMMC-7721; DOX loading into the nanocomposites then induced selective cytotoxicity against lung cells after 24-h exposure. Interestingly, DOX-loaded nanocomposites did not induce cell death in the non-cancerous L929 cell line. These findings suggest that the modified GO materials are strong potential candidates for targeted anticancer drug delivery systems [179]. Normal fibroblast (3T3) and liver cancer cells (HepG2) were treated with different concentrations of GO, gallic acid (GA)-functionalized GO (GOGA), or GA for 72 h. Surprisingly, the GOGA nanocomposite showed an inhibitory effect on cancer cell growth without affecting normal cell growth [180]. An rGO/gold nanorod (AuNR)/hydroxyapatite (HA) nanocomposite carrying 5-FU showed robust, selective targeting and penetrating efficiency against HeLa cells due to the significant compatibility and nontoxicity of HA and showed excellent synergetic antitumor effects with minimum side effects through combined chemotherapy of 5-FU plus PTT by both rGO and AuNRs under NIR laser irradiation. This multicomponent system has potential application in biomedical technology [181]. Yuan and Gurunathan (2017) demonstrated that the potential cytotoxicity of rGO-AgNP nanocomposites in human cervical cancer cells was greater than GO, rGO and AgNPs. Particularly, the combination of CP and rGO-AgNPs enhances cytotoxicity, apoptosis and autophagy in HeLa cells [182].

### 2.7. Metallic Nanoparticles (MNPs)

**Figure 7:**
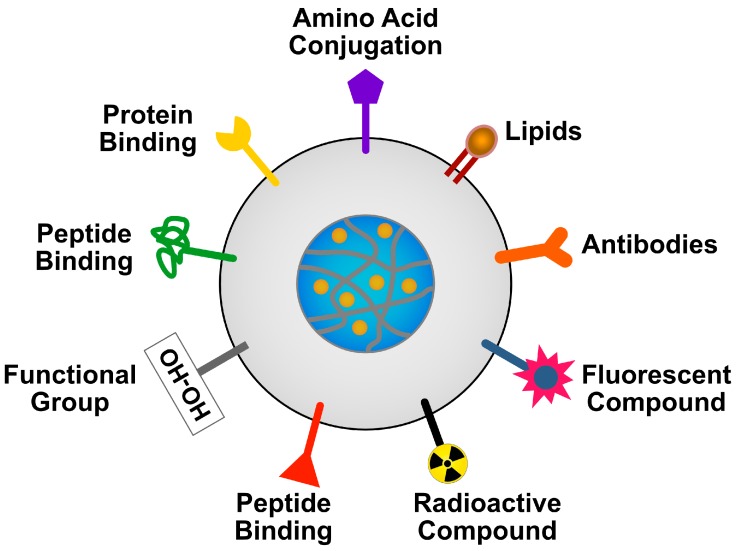
Different approaches to modify surfaces of nanoparticles (NPs) to target cancer cells.

Metallic NPs (MNPs) have been used widely in biomedical sciences and engineering. In order to use them for different biomedical applications, these MNPs can be synthesized and modified with various chemical functional groups [183]. MNPs have an average size between 1 and 100 nm. Based on size, they are classified into four types: MNPs (0D), metallic nanowires and rods (1D), metallic sheets and platelets (2D) and metallic nanostructures (3D). MNPs exhibit unique shape- and size-dependent optical properties. Among several MNPs, Ag, Au, palladium, platinum, iron oxide, zinc oxide, nanoshells and nanocages play an important role in biomedical applications. Advancements in nanotechnology have also enabled us to modify the surface of NPs with different moieties including functional and chemical groups, biological molecules and radioactive agents according to target applications (Figure 7).

AgNPs are used as antibacterial, antiviral, antifungal, anti-inflammatory, anticancer and anti-angiogenic agents [184]. However, using AgNPs alone as an antimicrobial agent or anticancer agent provides minimal activity when compared to combined treatment. Therefore, the combination of AgNPs with an anticancer drug is essential to overcome chemoresistance and undesired side effects. For example, the chemotherapeutic drug 5-FU is cytotoxic to a large number of cells but its activity is quite low in many cancer cells. Interestingly, the combination of AgNPs and 5-FU increases the induction of apoptosis by leakage of LDH and causes cellular DNA fragmentation in baby hamster kidney (BHK21) and human colon adenocarcinoma (HT29) cell lines [185]. The Au-Ag nanorod combination offers selective and efficient photothermal killing of targeted tumor cells by addressing two important issues: requirement of low laser radiation instead of high power and specific recognition of individual tumor cell types without any prior information about the biomarkers of the cells [186]. A combination of AgNPs with ionizing radiation exhibited radio and thermo sensitivity on U251 cells. In the presence of AgNPs, both X-rays and heat could enhance the cellular uptake of AgNPs. As intracellular AgNPs accumulated, the apoptosis rate of U251 cells was enhanced. Furthermore, AgNPs significantly inhibited cancer cell proliferation when combined with hyperthermic conditions [187]. The combination of AgNPs and DOX could form stable complexes that strongly interacted with DNA. This suggests that AgNPs and DOX are capable of altering DNA structure in a way that transitions DNA conformation to an ordered and compact molecular form, which then leads to greater inhibition of cell proliferation observed in T47D and MCF7 cells compared to a single treatment with either DOX or AgNPs alone [188].

The combination of sanazole (AK) and DOX complexed with AgNPs (SN-AK-DOX) increases the potential anticancer activity by increasing cytotoxicity and apoptosis in Dalton’s lymphoma ascites tumor cells above that of treatment with either sanazole or DOX or AgNPs alone. Furthermore, tumor-bearing animals injected with SN-AK-DOX resulted in significant reduction in tumor volume and delay in tumor growth [189]. The combination of AgNPs and salinomycin inhibits cell viability and proliferation of ovarian cancer cells by inducing ROS generation and leakage of LDH. Further, the combination increases the level of MDA and decreases various antioxidant levels than does a single treatment [190]. Similarly, Yuan et al. (2017) reported that the combined treatment gemcitabine and AgNPs caused increased cytotoxicity and apoptosis in A2780 cells by oxidative stress and upregulation of pro-apoptotic genes and downregulation of anti-apoptotic genes. The combination of CP and GO-Ag nanoparticles produced significant apoptosis in human cervical cancer cells by the expression of apoptotic and autophagy genes and the combination induced the accumulation of both autophagosomes and autophagolysosomes, which was associated with the generation of ROS [182]. HDAC inhibitors are well known as anticancer agents by inhibiting cell viability and inducing apoptosis in a variety of cancer cells. Combination of an HDAC inhibitor such as trichostatin A with GO-Ag NPs induced DNA fragmentation and double-strand breaks and eventually induced apoptosis, all at higher levels than did a single treatment in human ovarian cancer cells [191].

AuNPs are known to be non-toxic and non-immunogenic and are widely used as efficient drug delivery agents to target tumors [192]. To overcome the side effects caused by anticancer drugs, Brown et al. (2010) designed a platinum-based anticancer drug that tethered the active component of the anticancer drug oxaliplatin to an AuNP using PEG [193]. The platinum-tethered NPs increased cytotoxicity, drug uptake and localization in the A549 lung epithelial cancer cell line and the colon cancer cell lines HCT116, HCT15, HT29 and RKO. AuNPs stabilized by L-aspartate combined with anti-cancer drugs DOX, CP and capecitabine decreased cellular proliferation rates in hepatocellular carcinoma cells and increased susceptibility of cancer cells to treatment. Smaller AuNPs with an average size of 2.8 nm conjugated to DOX profoundly inhibited the proliferation of B16 melanoma cells compared to DOX alone [194]. AuNPs potentiate the cytotoxicity of anticancer drugs such as cyclophosphamide in alpha human folate receptor-positive breast cancer cells by conjugating the GNPs to the cancer-targeting ligand, FA [195]. AuNP-mediated delivery of a proteasome inhibitor (such as bortezomib) effectively inhibited cell viability and enhanced the anticancer effect in adenocarcinoma cells through rapid internalization and the formation of endocytic vesicles. Hence, the combination of AuNPs and bortezomib increased cell toxicity when compared to using the drug alone [196]. DOX-loaded Au-coated superparamagnetic iron oxide NPs (SPIONs@Au) is a combined complex that efficiently releases drugs at acidic conditions. As a result, the cell viability and proliferation was efficiently inhibited in MCF-7 cells treated with SPIONs@Au [197]. The combination of DOX-Hyd@AuNP efficiently transported and released the drug intracellularly into cancer cells, which led to reduction of mammosphere formation capacity and their cancer initiation activity, eliciting markedly improved tumor growth inhibition in murine models [198]; AuNPs prevented CP-induced acquired chemoresistance and stemness in ovarian cancer cells and sensitized them to CP [199]. AuNPs enhanced the treatment response when combined with CP-based fractionated chemoradiation in triple negative breast cancer cells and tumor xenografts [200]. CP and AuNPs synergistically induce cytotoxicity in the MM200 cell line by facilitating CP internalization in the presence of GNPs. In addition, microwave exposure further improves the efficacy of CP therapy in the presence of GNPs on MM200 cells [201].

Palladium NPs (PdNPs) have unique properties in characteristics such as catalytic, optical, high surface-to-volume ratio and high surface energy, nano architectonics and phototherapeutic and anticancer activities [202,203], thus allowing them to be useful in several biomedical applications. However, the usage of PdNPs is limited compared to Ag and Au NPs. Having an average size of only 10 nm, reduced-GSH-coated Pd nanosheets showed excellent renal clearance, prolonged blood circulation, accumulation in tumors and no apparent toxicity [204]. DOX-loaded mesoporous silicaNPs decorated with Pd nanosheets exhibited a significant synergistic effect in the presence of photothermal and chemo treatments [205]. Shi et al. (2013) reported that in tumor-bearing mice treated with an intratumoral injection of mesoporous silica-coated Pd@Ag NPs covalently loaded with the Chlorin e6 photosensitizer in the presence of 660-nm and 808-nm laser treatment, the tumors were completely destroyed [206]. Similarly, tetrasubstituted carboxyl aluminium phthalocyanine used as a photosensitizer allowed efficient PD and PT effects under a single 660-nm laser irradiation [207]. Human cervical cancer cells treated with either TSA or PdNPs showed a dose-dependent response in cell viability. However, the combination of TSA and PdNPs showed a more dramatic inhibitory effect on cell viability, compared to either TSA or PdNPs alone. The combination of TSA and PdNPs produced the significant cytotoxicity through oxidative stress, loss of mitochondrial membrane potential (MMP), activation of caspase-3/9 activity and expression of pro- and anti-apoptotic genes [208]. Similarly, the combination of tubastatin A and PdNPs exhibited more pronounced apoptosis in human breast cancer cells compared to either treatment alone through significant inhibition of HDAC activity and modulation of various cellular and biochemical changes. Nanohybrids, containing the anticancer drug PTX and the photothermal agent palladium phthalocyanine, increased permeation of the cancer cells and increased the retention of the drugs; therefore, the tumor was eradicated efficiently by reducing the side effects of the loaded PTX [209].

Platinum-based NPs used for combination therapy to promote synergetic efficacy and to overcome the resistance of platinum drugs and platinum-based NPs are able to localize to the tumor where it can prevent tumor recurrence. Furthermore, the combination of platinum drugs and imaging agents in a single platform allows diagnosis of cancer and real-time monitoring of the distribution of drug-loaded NPs inside the body and tumor [210]. The combination of platinum NPs with irradiation by fast ions enhances lethal damage in DNA [211]. The encapsulation of PtNPs in liposomes (LipoPtNPs) caused an approximately 2.4-fold higher rate of DNA damage in comparison to CisPt, LipoCisPt, or PtNPs alone in human foreskin fibroblast through inhibition of DNA replication and ultimately the treatment affected the secondary structure of DNA, leading to high cytotoxicity [212].

Magnetic nanoparticles (MNPs) are composed of a metallic core stabilized and functionalized by the addition of an outer shell with conjugated functional groups. MNPs are normally smaller than 100 nm and can be synthesized from any material characterized by some degree of magnetism [213,214,215]. MNPs are more stable and responsive as single-domain structures deployed in conditions that exceed the Curie temperature. Superparamagnetic nanoparticles (SPNs) has various salinet features such as low remanence and coercivity, coupled with a high magnetic susceptibility [216,217]. MNPs exhibited stable in chemically and physically challenging microenvironments such as hypoxic tumor conditions. MNPs utilized for several biomedical applications including MRI, promoting the accumulation and deposition of biotherapeutic compounds such as genes and peptides in rewarding microniches and mediating the destruction of cancer cells. In addition, MNPs can be targeted to specific tissues, including cancer cells and in the field of magnetic hyperthermia. These features facilitate to use MNPs as versatile and adaptable tools in various biomedical applications. However still it has some limitations to the use of MNPs in the diagnosis and treatment of cancer and infectious diseases.

## 3. State of the Art of Combination Therapy and Limitations

Cancer is the most common malignant disease both men and women worldwide but the current drug therapy has limitations. To overcome the limitations, a new avenue of nanotechnology called nanomedicine provides alternative strategy for cancer treatment. Although varieties of drugs are available in the market, these products were originally designed for generic anticancer purpose and not specifically for specific type of cancer treatment. Recently, number of novel and promising nanobased nanotherapeutic strategies and devices were developed and particularly nanotechnology provides to develop nanomedicine that is more tailored for specific and individual type of cancer in cancer with more specificity with anticancer effect and drug resistance reversal effect. It is expected that more products will enter with high efficacy and less side effects in clinical arena. However, caution must be exercised in adopting these advanced strategies. The biggest issue is about the increased complexity of the nanoformulation. The technocrats of nanotechnology are essential to provide considerable evidence for nanoformulations having clinically more effective, sufficiently stable and cost-effective. Finally, the most important aspects of nanomedicine strategies should conduct strong collaboration with experts in variety of field dealing with cancer therapy such as nanoformulations, toxicology, pharmacokinetics, toxicology, cell and molecular biology, immunology and oncology.

**Figure 8:**
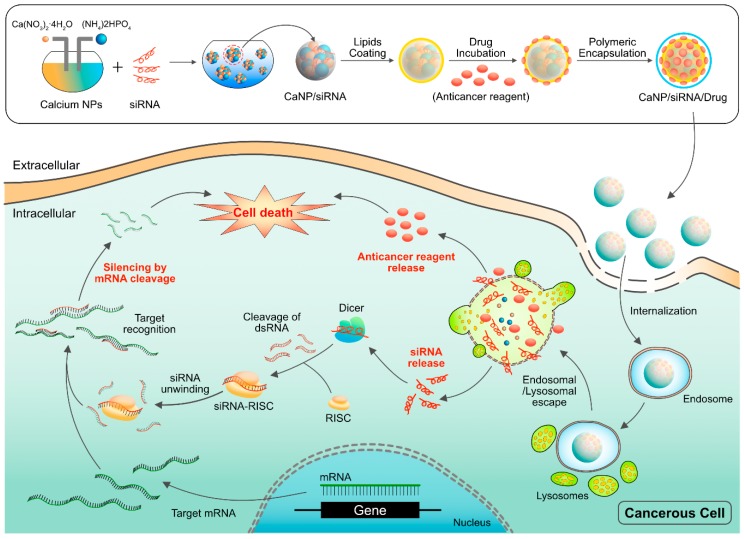
Combined nano-therapy strategy of CaP nanoparticles containing siRNA and anticancer reagent and their therapeutic effect in cancerous cell. CaP nanoparticles which were assembled with siRNA targeting specific genes is coated with lipids, followed by co-incubation with anticancer drug. The obtained CaNPs/siRNA/DOX are encapsulated by polymers. Internalized CaNPs/siRNA/DOX into cancerous cell are dissolved in endosomal pH through endosomal/lysosomal escape and then released to cytoplasm. Then, siRNA is processed by dicer and RISC system in cytosol and recognize target mRNA to silence the gene expression. Simultaneously, anticancer drug released functions regarding its biological mechanism. Ultimately, both are purposed to induce cell death.

**Figure 9:**
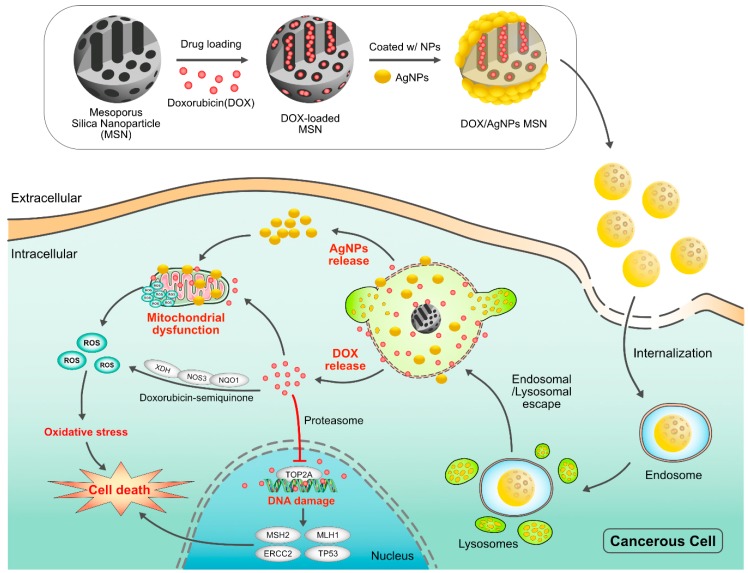
Combined nano-therapy strategy of MSN containing DOX and AgNPs in cancerous cell. MSNs are loaded with DOX then coated with AgNPs. The obtained DOX/AgNPs/MSN is internalized into cancerous cell via endocytosis and then released to cytosol by endosomal pH through endosomal/lysosomal escape. Released AgNPs resulted in mitochondrial dysfunction by inducing ROS production in mitochondria. DOX released accelerate ROS production, followed by induce oxidative stress in cytosol via DOX semiquinone. DOX also is intercalated into DNA that results in DNA damaging by poisoning of topoisomerase II. Both of AgNPs and DOX finally induce cancer cell death through oxidative stress by increasing ROS and DNA damaging in nucleus. Black colored indicates direction of pathway and red color indicates blocking function.

Calcium Phosphate Nanoparticles (CaPN) and Mesoporous Silica Nanoparticles (MSN) are considered idle candidates for combined drug delivery to target cancer cells due to their biocompatibility, bioactivity, pH responsive and porous nature [218,219]. Different strategies for combine cancer therapy by using these nanoparticles is explained graphically in Figure 8 and Figure 9. But CaPN have limitation to load limited amount of drug, initial burst release and short-term release. This problem of CaPN can be replaced with MSN which has high drug loading capacity due to porous nature and drug release time can be controlled by functionalizing the gatekeeper system over MSN (Figure 9).

To achieve a perfect candidate for drug delivery medical and materials scientists are continuing various researches with advanced engineering approaches therefore it is expected that more products will enter with high efficacy and less side effects in clinical arena. However, caution must be exercised in adopting these advanced strategies. The biggest issue is about the increased complexity of the nanoformulation. The technocrats of nanotechnology are essential to provide considerable evidence for nanoformulations having clinically more effective, sufficiently stable and cost-effective. Finally, the most important aspects of nanomedicine strategies should conduct strong collaboration with experts in variety of field dealing with cancer therapy such as nanoformulations, toxicology, pharmacokinetics, toxicology, cell and molecular biology, immunology and oncology.

## 4. Conclusions and Future Perspectives

Currently available conventional cancer therapeutic strategies suffer from severe limitations such as bio-distribution, insufficient targeting by the therapeutic agents, poor solubility, poor oral bio-availability, low therapeutic indices, dose-limiting toxicity to healthy tissues and most importantly, almost invariably an emerging drug resistance. Recent clinical and preclinical data showed that combination therapy exhibited potential applications in cancer. NPs are multitalented platforms used to act with anticancer drugs to enhance the therapeutic performances in cancer cells through maintaining drug stability and providing bio-availability, long-term retention, preferential accumulation of drugs in tumor sites via enhanced permeation and retention effects, reduced side effects and an increased therapeutic index. Over the last decade, nanoparticle systems have been successfully harnessed direct anticancer agents or for co-delivery of multiple anticancer agents. The use of NPs in combination with other anticancer agents regulates multiple pathways involved in the various stages of cancer cell, including growth, progression, metastasis and drug resistance. The NP-constituted nanomedicine leads to an unsurpassed opportunity to move forward for the treatment of a variety of diseases, including cancer. The unique properties of NPs include size, large surface-to-volume ratio, the ability to encapsulate a variety of drugs and tunable surface chemistry. Lipid bi-layered vesicles called liposomes are frequently used due to their efficiency, bio-compatibility, non-immunogenicity and their abilities to enhance solubility of chemotherapeutic agents and encapsulate a wide array of drugs. The disadvantages of lipoasomes are low solubility, short half-life, production cost and oxidation of phospholipids. Polymeric nanoparticles are biodegradable, significant physical and mechanical properties and the disadvantages of polymeric nanoparticles exhibited the drug release is typically biphasic and uncontrolled.

PMs can specifically target tumor sites by passive as well as active mechanisms and also have beneficial inherent properties including size, stability in plasma, longevity in vivo and EPR effects through enhanced penetration and uptake of anticancer agents. Polymeric micelles are uniquely suited for multi-drug delivery due to selectivity, synergistic and safe anticancer drug combinations. The disadvantages of PMs are size and intratumoral penetration. Dendrimers are nanostructures that show potential abilities in entrapping and/or conjugating the high molecular weight hydrophilic/hydrophobic entities and have been used as promising candidates in drug discovery and clinical applications due to high ratio of surface groups to molecular volume and major limitations are toxicity. CNTs, carbon NPs, ND and fullerenes have been used as nanocarriers and are strong tools in the biomedical fields as well as for non-invasive cancer therapy. Graphene and graphene-related family members have been used as drug-delivery vehicles for cancer therapy and appear to be novel therapeutic agents for performing on-demand chemotherapy coupled with PTT or PTD. The use of carbon nanosystems are having numerous benefits in combination therapy including high stability, large surface area and biocompatibility. The major disadvantages of various carbon nanosystems are protein opsonization, insolubility and strong tendency to aggregate.

MNPs are alternative agents to overcome problems related to conventional chemotherapy and serve a beneficial and powerful role in cancer therapy by providing better targeting, gene silencing, drug delivery and also because they can act as cytotoxic agents either alone or with anticancer drugs. The major advantages of using MNPs are easy functionalization, conjugation with variety of antibodies, ligands and drugs of interest. These salient features lead opening a wide range of potential applications in the area of biomedical engineering. However, MNPs are shows several limitations such as penetration, tissue non-specificity and cost of materials.

Combination therapy provides synergistically enhanced therapeutic outcomes without added toxicity, enhanced efficacy and overcomes drug resistance to elicit sustained treatment responses in cancer patients. In addition, variety of advantages characters involved in combination therapy using nanoparticles and anticancer agents such as drug payloads, prolonged circulation in the bloodstream, reduced frequency of dosage required for therapeutic efficacy, uniform, sustained drug release kinetics. Although nanoparticles mediated, combination therapy has several features it has some limitations/disadvantages such as lack of relevant pre-clinical models for testing their targeting efficiency and lack of collaboration between theoretical and experimental scientists, engineers, medical doctors, pharmaceutical and biotechnology industries. At present, there is a limited nanoproduct are available for clinical use and we have to consider the cost of nanodrug compared to available cancer drugs. The costs of these nanocombinations are likely to be significantly higher than the collective cost of the individual drugs combined. Therefore, these kind of questions need to be addressed using a multidisciplinary approach with collaborations between academia, the pharmaceutical industry and the regulatory bodies involved to ensure that nano-combination therapy delivers on its promise of better treatment outcomes while severely reducing morbidity thus improving the quality of life in cancer patients. In summary, combination therapy provides an attractive strategy for treatment of a variety of cancers to help overcome drug resistance, low efficacy and high-dose-induced systemic toxicity. However, tackling the challenges will require enormous effort. NP-mediated combination therapy holds great promise for successful clinical translation of nanomedicine treatments, which will provide unique benefits to cancer patients. Nevertheless, the future studies are warranted to address safety, biocompatibility, easy availability and particularly study should focus on toxicity. The most important aspect of future studies is the requirement to address a variety of animal models and possible clinical studies on humans need to be performed to substantiate their use in cancer therapy.
